# Current and future trends in periodontal tissue engineering and bone regeneration

**DOI:** 10.20517/2347-9264.2020.176

**Published:** 2021-01-08

**Authors:** Matthew Galli, Yao Yao, William V. Giannobile, Hom-Lay Wang

**Affiliations:** 1 Department of Periodontics and Oral Medicine, University of Michigan School of Dentistry, Ann Arbor, MI 48109, USA.; 2 Biointerfaces Institute, North Campus Research Complex, University of Michigan School of Dentistry, Ann Arbor, MI 48109, USA.; 3 Harvard School of Dental Medicine, Boston, MA 02115, USA.

**Keywords:** Tissue engineering, periodontics, bone regeneration, wound repair/healing, regenerative medicine, dental implants

## Abstract

Periodontal tissue engineering involves a multi-disciplinary approach towards the regeneration of periodontal ligament, cementum and alveolar bone surrounding teeth, whereas bone regeneration specifically applies to ridge reconstruction in preparation for future implant placement, sinus floor augmentation and regeneration of peri-implant osseous defects. Successful periodontal regeneration is based on verifiable cementogenesis on the root surface, oblique insertion of periodontal ligament fibers and formation of new and vital supporting bone. Ultimately, regenerated periodontal and peri-implant support must be able to interface with surrounding host tissues in an integrated manner, withstand biomechanical forces resulting from mastication, and restore normal function and structure. Current regenerative approaches utilized in everyday clinical practice are mainly guided tissue/bone regeneration-based. Although these approaches have shown positive outcomes for small and medium-sized defects, predictability of clinical outcomes is heavily dependent on the defect morphology and clinical case selection. In many cases, it is still challenging to achieve predictable regenerative outcomes utilizing current approaches. Periodontal tissue engineering and bone regeneration (PTEBR) aims to improve the state of patient care by promoting reconstitution of damaged and lost tissues through the use of growth factors and signaling molecules, scaffolds, cells and gene therapy. The present narrative review discusses key advancements in PTEBR including current and future trends in preclinical and clinical research, as well as the potential for clinical translatability.

## INTRODUCTION

**Figure 1.**
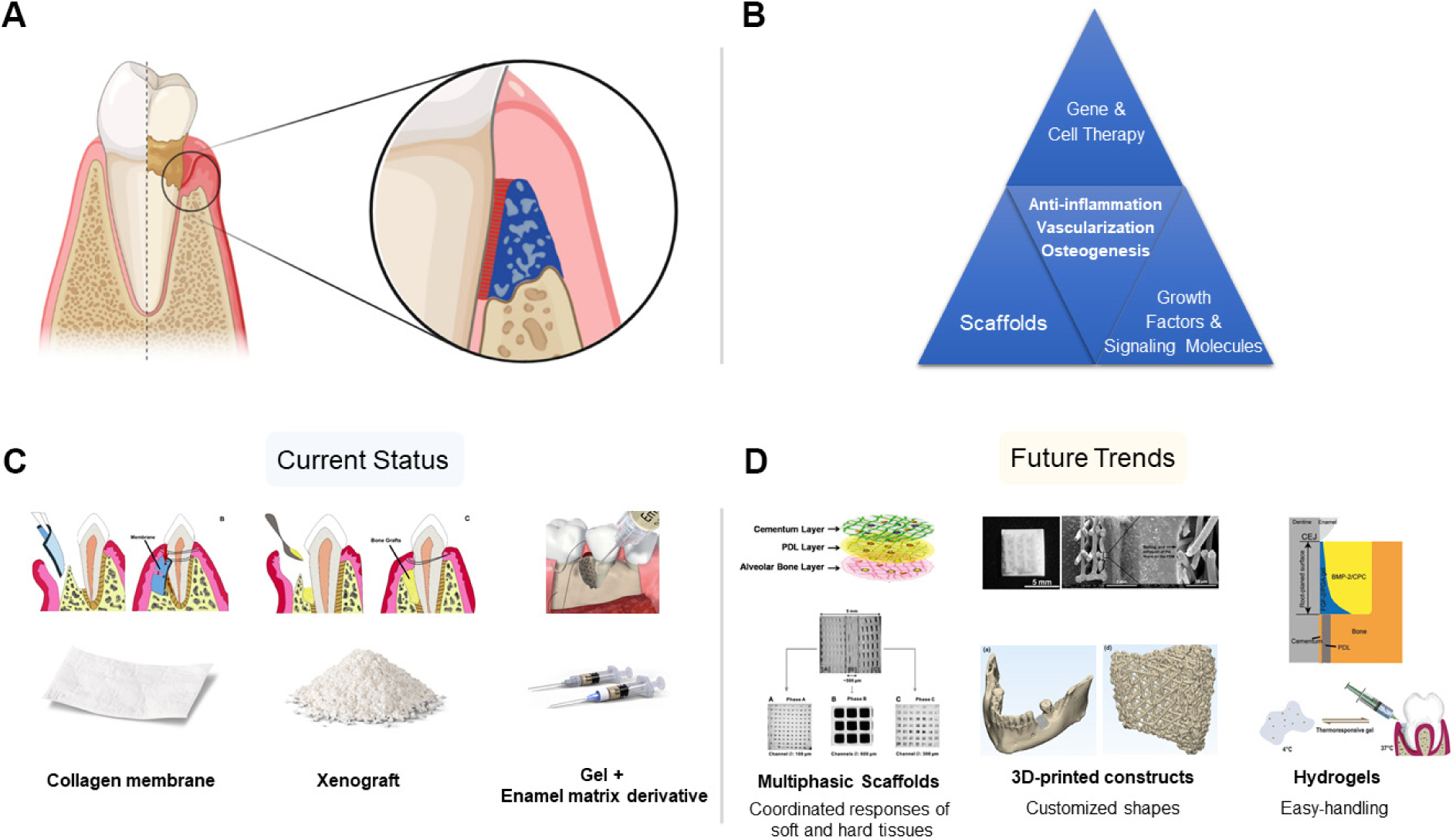
Apical migration of the supracrestal tissue attachment, formation of periodontal pockets, and destruction of PDL and alveolar bone are sequalae of periodontitis. After decontamination of the defect, periodontal tissue engineering-based treatment strategies can be implemented to regenerate the periodontium, and restore structure and function (A); treatment approaches in periodontal tissue engineering employ gene therapy, cell therapy, scaffolds, and growth factors/signaling molecules alone or in combination. Successful regenerative outcomes rely on controlling inflammation, and promoting vascularization and osteogenesis (B); three commonly used biomaterials in everyday clinical practice that are considered components of tissue engineering-based approaches are: collagen membranes, xenograft, and growth factors/signaling molecules (i.e., enamel matrix derivative) (C); biomaterials which may enter clinical practice in the future include multiphasic scaffolds, 3D-printed constructs and hydrogel delivery systems (D). PDL: periodontal ligament

The management of periodontal and peri-implant diseases constitutes a significant healthcare burden with the potential to greatly improve the quality of life of affected patients^[1]^. These microbially-mediated inflammatory diseases are destructive in nature and culminate in progressive loss of tooth-supporting alveolar bone. Besides periodontitis and peri-implantitis, orofacial soft and hard tissue deficiencies can also be caused by post-extraction atrophy, trauma, tumor resection, and congenital or developmental conditions such as cleft lip and palate^[2]^. The field of tissue engineering/regenerative medicine (TE/RM) has emerged to manage these clinical scenarios, with the goal of replacing and regenerating lost or damaged tissues to restore normal function and structure [Figure 1A]^[3]^. Craniofacial tissue engineering employs a wide range of approaches generally centered on conduction and induction of host cells, as well as cell transplantation and gene therapy^[4]^. Periodontal regeneration is an important component of this vast field and involves the reconstitution of cementum, periodontal ligament (PDL) and alveolar bone around teeth^[5]^. Bone regeneration applies specifically to the treatment of peri-implant hard tissue deficiencies, alveolar ridge defects in need of augmentation for future implant placement and sinus floor augmentation^[6]^. Some of the major clinical challenges encountered in periodontal tissue engineering and alveolar bone augmentation include developing strategies to overcome masticatory forces, the avascularity of the tooth and implant surfaces, and the microbial contamination inherent to working in the oral cavity. In order to tackle the complexity of regenerating the periodontium which is composed of numerous cell types and tissue interfaces, therapeutic approaches in TE/RM must draw from many fields of research spanning both the basic science and clinical realms. Knowledge from molecular and cell biology, medicine, manufacturing, materials science and nanotechnology must be amalgamated in a multi-disciplinary manner to develop successful periodontal and peri-implant tissue engineering treatment approaches^[7]^.

In the context of periodontal regeneration, TE/RM has evolved significantly over time beginning early on with the concept of guided tissue regeneration (GTR). Melcher^[8]^ was the first to postulate that the specific cell types that initially repopulate the root surface following periodontal surgery will determine the nature of the new attachment and whether healing proceeds by repair or regeneration. The biologic rational for the GTR treatment concept is based on the implementation of cell-occlusive barrier membranes to selectively exclude relatively rapid epithelial and fibroblastic downgrowth, while promoting repopulation of defect sites with slower migrating cells from the periodontal ligament, bone and cementum^[2]^. Guided bone regeneration (GBR) was developed for the regeneration of osseous defects based on the principles underlying GTR, therefore also incorporating the use of barrier membranes to mechanically exclude soft tissue ingrowth from the defect site to promote repopulation with osteogenic progenitor cells^[9]^. GBR may be utilized for regeneration of critical size maxillofacial deficiencies, peri-implant bone defects and also for post-extraction alveolar ridge augmentation.

The major components of current tissue engineering-based treatment approaches include signalling molecules/growth factors, scaffolds, and cells with particular focus on promoting osteogenesis, angiogenesis, as well as controlling inflammation [Figure 1B]^[10]^. It is important to note that these approaches can be used either alone or in combination with one another. Since the initial development of GTR and GBR, significant advancements have been made surrounding the implementation of biologics, scaffolds and gene therapy, with the aim of modulating the healing response to facilitate regenerative outcomes [Figure 1C and D]^[11]^. One of the major aims of recent research in periodontal tissue engineering and bone regeneration (PTEBR) is to develop therapeutic modalities capable of temporospatial recruitment and direction of host cells in a manner which promotes regeneration and healing. In addition to determining the appropriate biologics and growth factors to utilize for regenerative treatments, it is also critically important to elucidate the optimal doses and delivery methods.

The present review provides an overview of important advancements in PTEBR from the perspective of recent preclinical and clinical trials, discussing their implementation and clinical translatability. In particular, the present review will focus on current trends in PTEBR pertaining to signalling molecules/growth factors, scaffolds, as well as gene and cell therapy [Figure 1]. Cell-free therapy involving stem cell-derived extracellular vesicles will also be discussed.

## GROWTH FACTORS AND SIGNALING MOLECULES

### Growth factors and signaling molecules

Growth factors and signalling molecules used in PTEBR are proteins with the capacity to promote chemotaxis, proliferation, differentiation, extracellular matrix synthesis and angiogenesis^[12]^. The biologic functions of these molecular mediators vary widely, but their selection as candidates for regenerative therapy is based on their important roles in periodontal tissue development and wound healing. Promising results from preclinical and clinical trials led to the subsequent introduction of various growth factors into the commercial market for the purposes of periodontal, and peri-implant soft and hard tissue regeneration^[13]^. The present section focuses largely on growth factors that are commonly studied in the literature and have also been approved for clinical treatment. This section aims to provide the reader with an overview of mechanisms of action, indications, as well as preclinical and clinical evidence supporting the implementation of growth factors and signalling molecules in periodontal and peri-implant tissue engineering-based treatment approaches [Table 1].

### Enamel matrix derivative

Enamel matrix derivative (EMD) is composed of a combination of peptides harvested from immature enamel of developing porcine tooth buds, and was the first Food and Drug Administration (FDA)-approved biologic for periodontal regeneration^[12]^. Enamel matrix derivative contains a mixture of enamel matrix proteins within an aqueous solution of propylene glycol alginate (PGA), permitting application via a syringe due to its shear-thinning properties^[14]^. During tooth development, enamel matrix proteins secreted by Hertwig’s epithelial root sheath (HERS) play an important role in odontogenesis. Hammarström *et al.*^[15]^ demonstrated that amelogenin is the major enamel matrix protein expressed in human teeth during root formation, and in response to EMD exposure, hard tissue formation could be stimulated resulting in tissues histologically similar to extrinsic acellular cementum. These results were among the first to show the potential importance of EMD proteins during cementogenesis. Although early *in vivo* studies demonstrated promising results, in later preclinical and human studies, EMD has inconsistently induced acellular cementum formation leading to some questions over the precise mechanism of action^[16]^.

A review of *in vitro* investigations of EMD conducted by Grandin *et al.*^[17]^ indicated that in addition to enhanced PDL and osteoblastic gene expression, protein synthesis, mitogenesis and differentiation, EMD could also be involved in angiogenesis and inhibition of epithelial proliferation. Additionally, it was found that the regenerative capacity of EMD was not completely matched by full-length amelogenin, shorter amelogenin or ameloblastin peptides. These findings point towards a synergistic effect amongst amelogenin and non-amelogenin peptides found within EMD in a ratio approximating 9:1. Further studies are needed to delineate the biologic effects of EMD on osteoblastic and cementoblastic mesenchymal cells in order to develop a more precise understanding of mechanism of action.

In a systematic review, Nibali *et al.*^[18]^ compared clinical and radiographic outcomes after regenerative surgery and open flap debridement (OFD) for the treatment of deep (≥ 3 mm) intrabony periodontal defects. Both GTR and EMD were superior to OFD alone in terms of clinical attachment level gain. Although the use of EMD was associated with higher radiographic bone fill compared to OFD alone, no statistically significant differences in clinical and radiographic outcomes were found between EMD *vs.* GTR or EMD *vs.* bone filler alone. The authors concluded that EMD and GTR with absorbable membranes are the gold standard for treatment of deep intrabony periodontal defects. Additionally, a recent review by Tavelli *et al.*^[19]^ on biologics-based regenerative approaches for periodontal soft tissue engineering found that consistent high-quality evidence supports the adjunctive use of EMD for root coverage procedures. Human clinical studies have demonstrated promising results for the adjunctive use of EMD during surgical treatment of peri-implantitis^[20,21]^, finding increased bone levels after 12 months and decreased prevalence of gram negative microbes^[22]^. Despite these positive findings, the amount of studies investigating the use of EMD for stimulating new bone formation is limited^[23]^.

Overall, EMD has shown similar regenerative outcomes relative to the use of membranes and bone filler biomaterials, and is simpler to use, resulting in fewer post-operative complications^[24]^. Further research is needed to investigate the use of EMD for treatment of peri-implant defects, sinus floor augmentation and alveolar ridge augmentation.

### Recombinant human platelet-derived growth factor-BB

Recombinant human platelet-derived growth factor-BB (rh-PDGF-BB) is one of the most studied growth factors implemented in periodontal tissue engineering. Since its introduction in the late 1980s^[25]^, rh-PDGF-BB has become commercially available for use in the form of Growth-Factor Enhanced Matrix (GEM) 21S (Lynch Biologics), which utilizes β-tricalcium phosphate (β-TCP) as a scaffold^[26]^. PDGF is also one of the most abundant growth factors found in platelet rich plasma (PRP)^[27]^ and plasma rich in growth factors (PRGF)^[28]^, both of which are concentrates prepared from centrifugation of patient blood. Of note, the concentration of PDGF in GEM 21S is about 750-fold and 6,400-fold higher relative to the concentration of PDGF in PRP and PRGF, respectively^[29]^. The PDGF family is composed of four gene products capable of forming five different dimeric isoforms^[30]^. These isoforms include PDGF-AB, -AA, -BB, -CC, and -DD variants, each of which exhibit different affinities for PDGF ζ and β tyrosine kinase receptors^[31]^. PDGF-BB is known as the “universal PDGF” due to its ability to bind to all receptor isotypes. Boyan *et al.*^[32]^ conducted an *in vitro* investigation into the response of human PDL cells to the various PDGF isoforms, and discovered that the PDGF-BB isoform exhibited the strongest mitogenic and chemotactic effects. Stored in the ζ-granules of platelets and released upon activation, PDGF has also been shown to induce DNA synthesis, chemotaxis, as well as collagen and glycosaminoglycan production in fibroblasts^[33]^. In addition to platelets, activated macrophages and fibroblasts are also known to secrete PDGF^[34]^. PDGF has been demonstrated to stimulate chemotactic and mitogenic responses in mesenchymal cells (in particular PDL cells), in preclinical animal models^[35,36]^. *In vitro* analysis has also demonstrated that PDGF-BB is a powerful stimulator of stem cell marker expression and proliferation in mesenchymal stem cells (MSCs) isolated from human PDL tissues^[37]^. In addition, PDGF can modulate bone formation and regeneration through its actions on pericytes (cells of mesenchymal origin located on the perivascular surface believed to harbor populations of MSCs with osteogenic potential). Caplan and Correa^[38]^ presented evidence that PDGF plays an important role in mitotic expansion of pericytes and is a major molecular player in pericyte-MSC-osteoblast dynamics, acting as an osteoprogenitor cell mitogen and regulating activity towards osteogenic growth factors such as bone morphogenetic proteins (BMPs).

A recent meta-analysis of randomized clinical trials (RCTs) reported that treatment of periodontal osseous defects with rh-PDGF-BB resulted in significantly increased bone fill, lateral bone gain, clinical attachment gain and probing depth reduction, compared to control groups utilizing modified minimally invasive surgical techniques or bone filler biomaterial alone (β-TCP)^[39]^. In regards to root coverage procedures, no significant regeneration was found for recession defects treated with rh-PDGF-BB. Similarly, Tavelli *et al.*^[19]^ reported that current evidence is inconsistent regarding human clinical trials for treatment of recession defects using rh-PDGF-BB. Despite these findings, McGuire *et al.*^[40]^ demonstrated histologic regeneration after treatment of human recession defects with rh-PDGF-BB in conjunction with a β-TCP scaffold, whereas healing by repair (long junctional epithelium) was seen for sites treated with connective tissue grafting. In terms of GBR, Santana and Santana^[41]^ compared rh-PDGF-BB delivered in a β-TCP/hydroxyapatite (HA) carrier with autogenous block grafting and reported that no significant differences were observed in the amount of bone regeneration or the need for further grafting. Treatment of extraction sockets with rh-PDGF-BB exhibited no significant effect on healing^[42]^.

In summary, rh-PDGF-BB has demonstrated efficacy in the promotion of periodontal hard and soft tissue regeneration. Applications for use of PDGF to promote bone regeneration in extraction sockets, alveolar ridge regeneration and sinus floor augmentation are promising, but will benefit from more expansive RCTs to evaluate the efficacy of PDGF^[43]^.

### Fibroblast growth factor-2

Fibroblast growth factor (FGF)-2, also known as basic fibroblast growth factor, possesses strong mitogenic properties and has important roles in wound healing, granulation tissue formation, angiogenesis and tissue remodelling^[44]^. Heparin/heparin sulfates are essential for mediating FGF bioactivity^[45]^. When bound to heparin, the degradation rate of FGF-2 is significantly reduced and mitogenic activity is greatly enhanced^[46]^. Heparin sulfate proteoglycans also act as cofactors for FGF2-FGF receptor interactions, allowing for activation of downstream signalling pathways^[47]^.

In the context of PTEBR, FGF-2 has been utilized for the treatment of intrabony periodontal defects, peri-implant defects, improving implant osseointegration and GBR. A systematic review of human clinical trials by Khoshkam *et al.*^[48]^ found significantly greater bone fill compared to control groups for the treatment of intrabony defects, with pre-clinical evidence to support the induction of newly formed cementum, PDL and alveolar bone^[49,50]^. Another systematic review of RCTs by Li *et al.*^[39]^ found significantly greater bone fill using FGF-2 for treatment of intrabony periodontal defects compared to control groups, most of which used hydroxypropylcellulose alone. A dose-dependent effect was demonstrated, with 0.3% formulations of FGF-2 exhibiting the highest bone fill percentage and linear bone gain relative to formulations of 0.1% and 0.4%, for which there were no significant differences from controls. FGF has also demonstrated efficacy in promoting osseointegration in implants with low primary stability^[51]^. Preclinical studies have demonstrated promising results for GBR-based applications at edentulous sites and implants. Hosokawa *et al.*^[52]^ showed in a canine study that FGF-2 delivered in collagen minipellets accelerated bone regeneration in alveolar defects treated with GBR, resulting in significantly greater histometric bone gain. FGF-2 has also been the subject of numerous studies investigating combinational and sequential delivery, most often in conjunction with BMP-2. Wang *et al.*^[53]^ showed in a preclinical animal study that the combination FGF-2 and BMP-2 exhibited a synergistic effect in promoting ectopic bone formation. In addition, there is evidence to support the notion that sequential delivery of FGF-2 followed by BMP-2 is more efficient in terms of inducing differentiation of osteogenic progenitor cells than delivery of either agent alone^[54–56]^. These findings highlight the importance of conducting time-sequencing studies for therapeutic strategies involving multiple growth factors.

Overall, clinical evidence supports the safety and efficacy of using FGF-2 for treating periodontal intrabony defects^[57]^, but future clinical trials are needed to validate the use of this growth factor in the context of peri-implant and bone regeneration.

### Bone morphogenetic proteins

Bone morphogenetic proteins (BMPs) are the largest subfamily of the transforming growth factor-β (TGF-β) superfamily of growth factors^[58]^. BMP signal transduction is mediated by a cytoplasmic protein family called SMADS (specifically, SMAD-1, −5, and −8)^[59]^. Currently, fourteen BMPs have been identified, with BMP-2, 4, 5, 6, 7, and 9 demonstrating osteoinductive potential^[60]^. Of these, BMP-2 and −7 have been the most extensively studied for periodontal tissue engineering purposes. BMPs are a group of proteins responsible for guiding differentiation of mesenchymal cells into bone and bone marrow cells^[61]^, and have been demonstrated in preclinical studies to stimulate chemotaxis, survival and osteogenic differentiation of bone marrow mesenchymal stem cells (BMSCs)^[62]^.

Although the use of BMPs in periodontal regeneration has shown successful results in the treatment of intrabony and furcation defects^[63]^, complications such as ankylosis^[64]^ and root resorption have also been reported^[65]^. As a result, BMPs are currently indicated for implant site preparation during ridge preservation and sinus floor augmentation procedures^[26]^. rhBMP-2, commercially available as Infuse Bone Graft (Medtronic), is FDA approved for sinus augmentation and alveolar ridge preservation, while BMP-7 is not FDA approved for periodontal or peri-implant applications^[66]^. For clinical applications, rhBMP-2 is administered in conjunction with an absorbable collagen sponge (ACS) carrier, which has been FDA-approved for this purpose^[23]^.

Park *et al.*^[67]^ conducted a canine study evaluating the application of *ex vivo* adenoviral BMP-2 gene therapy using autologous periodontal ligament stem cells (PDLSCs) for the treatment of experimental peri-implantitis. In this study, two different models for inducing peri-implantitis were compared: immediate implant placement with simultaneous ligature placement, and delayed (3 months post-extraction) implant placement with delayed ligature placement. After treatment, > 70% bone fill was found in the delayed model, with 30%–40% in the immediate model. Other preclinical investigations have demonstrated positive effects of BMP-2 on peri-implant bone regeneration^[68,69]^. Interestingly, research has demonstrated greater bone regeneration after treatment with BMP-2/7 heterodimers compared to BMP-2 and −7 homodimers^[70]^, which suggests that combinatorial gene therapy has the potential to enhance osteoinductive potency. BMPs have also shown promising results for vertical bone regeneration in animal models, significantly enhancing the height and width of regenerated bone^[71]^.

A systematic review of human clinical trials conducted by Freitas *et al.*^[72]^ reported that sinus augmentation with autogenous bone resulted in significantly greater bone height compared to rh-BMP-2 in a collagen sponge carrier. However, a systematic review of clinical trials by Lin *et al.*^[73]^ demonstrated similar outcomes between sinus augmentation conducted with and without rh-BMP-2 in terms of vertical bone gain, bone density, percentage of residual graft material and percentage of vital bone. For alveolar ridge preservation, rh-BMP-2 maintained ridge height and enhanced ridge width in a dose-dependent manner. Additionally, a meta-analysis of human RCTs reported that treatment of extraction sockets, sinus floor augmentation, as well as cleft lip and palate alveolar reconstruction with rhBMP-2 did not result in statistically significant bone regeneration, with some results suggesting less new bone formation relative to control groups^[42]^. Interestingly, a split-mouth clinical study investigating sinus augmentation using BMP-7 reported significantly greater bone formation at the control side treated with bovine xenograft alone^[74]^.

In regard to GBR around implants, Jung *et al.*^[75,76]^ demonstrated that treatment of lateral bony defects with rhBMP-2, xenogenic bone and collagen membrane resulted in greater vertical defect fill, increased newly formed bone density, and a greater proportion of mature lamellar bone at regenerated sites after 6 months compared to GBR alone. These results were stable after 5 years follow-up and suggest that rhBMP-2 can potentially accelerate osseous healing in conjunction with GBR treatment. Regarding treatment of peri-implantitis defects, Sanz-Esporrin *et al.*^[77]^ found that GBR using rh-BMP-2 in conjunction with a bovine xenograft and collagen membrane did not result in significantly better regeneration compared to the control group. These results illustrate the difficulty of attaining successful regenerative outcomes in the face of bacterial contamination. However, there are some concerns regarding adverse events associated with BMPs in specific situations, including off-label indications. These include severe gingival swelling reported in children treated with BMP-2^[78]^, as well as structurally abnormal bone and increased inflammation pre-clinically^[79]^.

Although utilization of BMPs for alveolar ridge^[23]^ and sinus augmentation have demonstrated comparable outcomes to bone graft materials alone based on radiographic and clinical outcomes, other indications for the use of BMP-2 for bone regeneration in the oral cavity are warranted.

### Growth and differentiation factor-5

Growth and differentiation factor (GDF)-5 belongs to the TGF-β superfamily of proteins and bears a close structural resemblance to BMPs^[80]^. As a key regulator of skeletal, tendon and ligament morphogenesis, GDF-5 has also been shown to play important roles in odontogenesis^[81]^ and PDL development^[82]^. Preclinical studies have demonstrated enhanced bone formation in response to local delivery of GDF-5 in rat calvarial defects^[83]^, long bone healing^[84]^ and spinal fusion^[85]^. GDF-5 also plays important roles in wound healing and periodontal regeneration by regulating extracellular matrix metabolism^[86]^. Nakamura and coworkers demonstrated in a preclinical investigation that exposing human PDL cells to GDF-5 stimulated the synthesis of sulfated glycosaminoglycan and PDL cell proliferation, but not osteoblastic differentiation^[87]^. Moore *et al.*^[88]^ presented preclinical evidence for the use of GDF-5 in periodontal regeneration, showing increased new bone, cementum and PDL formation in small and large animal models. A review of both pre-clinical and clinical evidence by Lee and Wikesjö^[89]^ found that GDF-5 is a safe and effective candidate for periodontal regeneration and alveolar ridge augmentation, with one canine study showing greater alveolar bone and cementum regeneration compared to rh-PDGF for the treatment of intrabony defects^[90]^. For sinus augmentation, a RCT conducted by Stavropoulos *et al.*^[91]^ found similar amounts of newly formed bone for rhGDF-5/β-TCP compared to β-TCP and autogenous composite grafts.

Overall, GDF-5 used in conjunction with β-TCP has been demonstrated to stimulate periodontal regeneration in nonhuman primates^[92]^ and in humans^[93]^, but more clinical studies with larger sample sizes are needed to clarify these outcomes.

### Pro-resolving lipid mediators

Although scaffolds are vital to many tissue engineering strategies, implantation of these biomaterials can induce inflammatory responses capable of impairing integration and regenerative outcomes^[94]^. This limitation has led researchers to develop biomaterials with immunomodulatory properties capable of selectively influencing the immune response in a desirable fashion^[95]^. Pro-resolving lipid mediators such as resolvins and lipoxins are promising candidates for periodontal tissue engineering-based treatment approaches due to their ability to promote both inflammatory resolution and tissue regeneration^[96]^. Resolution of the acute inflammatory response is vital for attaining favorable regenerative outcomes^[97]^. Evidence from preclinical investigations in animal models demonstrate potential for pro-resolving lipid mediators such as resolvin E1 and lipoxin A_4_ in periodontal regeneration^[98]^. Lipoxins are lipid mediators produced by lipoxygenase enzymes in response to stimulation with prostaglandin E2 and D2. Cellular sources for lipoxins include epithelial cells, monocytes and neutrophils^[99]^. Van Dyke *et al*.^[100]^ evaluated the regenerative capacity of nano-proresolving medicines (NPRMs), a drug delivery agent consisting of membrane-shed vesicles containing a lipoxin analog (benzo-lipoxin A_4_; bLXA_4_), in the treatment of periodontal defects in a porcine model. Sites treated with NPRM-bLXA_4_ exhibited significantly more bone formation relative to bLXA_4_ alone, NPRM alone, and the negative control groups. Histology demonstrated newly formed bone and cementum coronal to the root notches with supracrestal connective tissue fibers parallel to the alveolar crest and anchored to the new cementum. Other preclinical studies have shown reduced systemic C-reactive protein and interleukin-1β after treatment of periodontitis with resolvins^[101]^, as well as increased fibroblastic proliferation *in vitro*^[102]^. Recently, Wang *et al.*^[103]^ demonstrated in a pre-clinical model that maresin-1 could accelerate both post-extraction osseous repair and also reduce post-operative pain. Clinical studies are needed to validate regenerative outcomes for pro-resolving lipid mediators. As of this writing, no human clinical trials have been conducted utilizing these mediators for periodontal tissue engineering and bone regeneration.

## ADVANCEMENTS IN SCAFFOLDS

Due to the hierarchical structure of the periodontium, successful periodontal regeneration remains challenging as it requires a series of coordinated responses across multiple soft and hard tissue interfaces^[104]^. One of the major tenets of tissue engineering is that biologics, including cells, proteins and genes, can be delivered via a degradable scaffold in order to promote regeneration. In general, scaffolds must fulfill four fundamental requirements: (1) form: able to match the geometry of complex 3D defects; (2) function: must transiently support functional and biomechanical demands during healing; (3) formation: should enhance regeneration; and (4) fixation: must readily interface and integrate with the surrounding tissues^[105]^. Tissue engineered scaffold constructs can provide a suitable microenvironment for recruited cells, optimize the beneficial effects of cell-based treatments, and enable the controlled release of biological cues such as growth factors. Thus, scaffolds are an inherently sound strategy for regenerating the complex anatomical structures comprising the periodontal tissues^[106–108]^.

Naturally derived biomaterials often exhibit similarities to endogenous ECM components and are generally designed to influence and modulate host cellular interactions in a desirable manner. Consequently, naturally derived scaffolds typically have strong biological characteristics and biocompatibility^[109]^. The most commonly used scaffolds in tissue engineering are bone filling biomaterials such as porous bone mineral substitutes (i.e., bovine-derived xenografts). Compared with biomaterials of natural origin, in the biomedical research field, synthetic polymers have distinct advantages. Synthetic polymers are easier to customize in terms of their micro- and macro-structural characteristics (i.e., pore size and degradation rate) and also possess a higher material stability than natural biomaterials such as collagen and gelatin. The most widely used synthetic polymers are polycaprolactone (PCL) and polylactic-co-glycolic acid (PLGA), both of which are FDA-approved biomaterials for drug delivery devices, sutures and adhesion barriers.

In this section, recent investigations on advanced scaffold constructs including multiphasic scaffolds [Table 2], 3D-printed scaffolds [Table 3] and hydrogels [Table 4] are summarized. Although scaffolds, growth factors, cell therapy and cell-free therapy represent different regenerative strategies, these technologies are often utilized in combination with one another in preclinical and clinical research, as well as in practice, in order to harness the unique advantages of each strategy.

### Multiphasic scaffolds

Multiphasic scaffolds are defined based on variations in architectural characteristics (such as porosity and pore organization) and chemical composition throughout a construct. They are often designed to resemble the structural organization as well as the cellular and biochemical composition of native tissues^[110]^. The design and fabrication of multiphasic scaffolds aiming to restore biomimetic functions to tissue-engineered hard and soft tissues has been recognized as a promising strategy in orthopedic tissue engineering, and has recently emerged in the field of periodontal tissue engineering within the last 10 years. Considering the complex structure of the periodontium and the interactions between multiple soft and hard tissues, Ivanovski *et al.*^[104]^ summarized key considerations in the design of multiphasic scaffolds for periodontal tissue engineering: (1) the compartmentalization of bone and periodontal attachment tissue formation that is integrated over time; (2) the promotion of cementum formation on the root surface; and (3) the formation of appropriately oriented periodontal ligament fibers that insert into newly formed bone and cementum.

Based on these principles, Vaquette *et al.*^[111]^ reported a biphasic tissue engineered construct for periodontal regeneration utilizing a solution electrospinning technique to fabricate a porous PDL compartment and a fused deposition modeling technique to fabricate a stiff bone compartment. This study demonstrated substantial cross-communication between the bone and periodontal compartments, and the newly-formed bone integrated with PDL-like tissue. However, this construct made solely from PCL did not provide any specific biochemical cues. Furthermore, appropriate biomechanical cues were missing because of the unmatched stiffness and porosity of the bone compartment in relation to natural bone. Triphasic nanocomposite scaffolds combined with three specific growth factors (CEMP-1 for the cementum layer, FGF-2 for the PDL layer, and PDGF for the bone layer) were reported by Sowmya *et al*.^[112]^. The results confirmed the formation of new cementum, fibrous PDL and alveolar bone with well-defined bony trabeculae in a rabbit maxillary periodontal defect. However, a major limitation was that the thickness and shape of the constructs were not modifiable, preventing a precise match between the dimensions of the defect (in terms of PDL space, cementum thickness and bone defect size) and the scaffold compartments.

Although multiphasic scaffold strategies seem to be well suited towards periodontal tissue engineering in terms of their ability to stimulate coordinated responses in both soft and hard tissues, there has been a scarcity of work on this topic^[113]^. To this end, as of 2020, no clinical studies have been published in the literature. Designing a multiphasic scaffold with strong cohesion between the different phases, sufficient surgical handling characteristics, and the ability to customize the morphology of the scaffold to adapt to clinical defects of varying shape and size are key aspects to consider in order to facilitate future clinical translation.

### 3D-printing

Over the past two decades, much effort has been applied towards creating porous constructs from the traditional freeze-casting and gas-foaming methods to the emerging wide variety of 3D-printing technologies that can be used to create ordered porosity and user-defined shapes^[114]^. Several 3D-printing techniques, such as 3D wax printing and fused deposition modeling, have been investigated to facilitate morphogenesis of the periodontal tissue complex.

Yeo *et al.*^[115]^ evaluated new bone formation of a PCL-TCP scaffold compared with autogenous block grafting for reconstruction of large dentoalveolar defects in a pig jaw model in 2011. Although histological examination revealed that the newly formed bone matrix was in direct contact with the scaffold, the bone volume fraction indicated that PCL-TCP scaffolds were approximately 51% as effective when compared to autografts. In 2012, Park *et al.*^[116]^ utilized 3D wax printing to fabricate a biphasic scaffold made of polyglycolic acid for the PDL compartment and PCL for the bone compartment. This ectopic periodontal regeneration model demonstrated some level of regeneration with functional orientation of newly formed periodontal fibers. In 2014, Lee *et al.*^[117]^ printed seamless scaffolds with region-specific microstructures consisting of three phases. It was shown that multiphasic scaffolds yielded aligned PDL-like collagen fibers that inserted into bone-like tissue as well as putative cementum matrix protein positive tissues. These conventional 3D-printing methods utilize fiber diameters between 100–200 μm and are thus incapable of fabricating high-resolution architectures down to the size of single cells (10–20 μm). Future studies should focus on improving the resolution of 3D printing methods in order to develop biomimetic scaffolds capable of reproducing and directing desirable cell-ECM interactions.

Although many previous studies have utilized standardized 3D-printed scaffolds for preclinical or clinical research, much effort has been made towards the fabrication and implementation of personalized patient-specific scaffolds^[118]^. Rasperini *et al.*^[119]^ published a landmark study describing the first reported human case involving treatment of a large periodontal osseous defect with a 3D-printed bioresorbable patient-specific scaffold. The treated site remained intact for 12 months following therapy, but became exposed at 13 months mainly due to the slow degradation rate and low porosity of the construct. In 2020, Bartnikowski *et al.*^[120]^ developed an accurate and reproducible workflow for fabrication of highly porous custom 3D-printed scaffolds for large volume alveolar bone regeneration. A higher degree of porosity has been associated with better cell ingrowth, nutrition infiltration, tissue integration, and can also potentially limit exposure of the construct^[119]^.

In conclusion, it is important that future 3D-printing studies take into account the unique requirements for regenerating the periodontal complex, especially the need for high-resolution constructs with optimized porosity and customizable shapes^[121]^.

### Gels and hydrogels

Compared to 3D scaffolds, a major advantage of injectable materials such as gels and hydrogels is their ease of adaption to irregularly shaped osseous defects via minimally invasive surgical techniques. *In situ* gels/hydrogels not only represent a novel concept of delivering drugs to patients in a liquid form, but can also achieve sustained release of bioactive molecules/drugs over a desired period of time. Different delivery systems based on polymers have been developed which are able to facilitate sustained release of drugs with numerous options for customized delivery^[122]^. In recent years, there has been an increasing interest in water-soluble polymers that are able to form gels after application to specific delivery regions within the human body. These so-called *in situ* gelling polymers are highly advantageous compared with other polymers as they can be easily applied in liquid form, which facilitates adaptation to defects with complex morphologies^[123]^.

A study conducted by Wang *et al.*^[124]^ developed a novel thermo-reversible hydrogel loaded with either doxycycline or lipoxin A_4_. This thermo-reversible construct was designed to be injected into a periodontal defect in a slightly cooled liquid state, to subsequently form a gel after absorbing body heat. Thermo-reversible hydrogels can be loaded with a wide range of antimicrobial or anti-inflammatory drugs for personalized periodontal applications.

In addition to the well-accepted drug delivery platforms created by gels and hydrogels, a novel concept of using gels/hydrogels as bioinks for 3D bioprinting in periodontal tissue engineering was tested by Thattaruparambil Raveendran *et al.*^[125]^ in 2019. They used a gelatin methacryloyl (GelMA) hydrogel and systematically investigated the influence of different printing parameters such as photoinitiator concentration, UV exposure, pressure and dispensing needle diameter on the viability of PDL cells contained within the 3D-bioprinted scaffold. This optimized bioprinting system is regarded as a major advancement towards the reproducible fabrication of cell-laden constructs for applications in periodontal and bone regeneration.

## GENE AND CELL THERAPY

### Gene therapy

For tissue regeneration, it is essential to trigger appropriate and precise cellular signals by growth factors to direct host cell populations in order to recapitulate endogenous regenerative differentiation potential^[10]^. The precise delivery of growth factors can be challenging as they rapidly degrade due to short half-lives and also diffuse into surrounding tissues. For example, in a porcine model, the half-life of PDGF-BB was demonstrated to be 4.2 h with 96% clearance occurring within 96 h^[25]^. Short half-lives and diffusion from the site of intended action are inherent properties of topical growth factor application, which typically occur in a single high-dose bolus^[126]^. This can lead to a burst release over a short time frame, which can possibly interfere with the bioactivity of other growth factors^[127,128]^. Gene therapy allows for sustained growth factor synthesis and secretion, working towards overcoming these limitations. Engineering cells through gene therapy to synthesize and secrete growth factors represents an alternative approach to controlling stem cell differentiation^[128]^, and allows cell-mediated production of proteins with authentic post-translational modifications and increased biological activity^[129]^.

Vectors used in gene therapy include plasmids, adenoviruses (Ad), lentiviruses, retroviruses, adeno-associated viruses (AAVs) and baculoviruses, each of which have their own advantages and disadvantages^[130]^. Dunn *et al.*^[131]^ implemented delivery of Ad-BMP-7 using a collagen matrix in a preclinical model in order to treat peri-implant osseous defects and reported that gene delivery began on day 1 and reached peak expression at day four. Gene treatment of dental implant fixtures with Ad-BMP-7 resulted in enhanced alveolar bone defect fill, coronal new bone formation and new bone-to-implant contact.

As higher viral transduction rates contribute towards an increased risk for host immunogenicity, and higher doses of virus can lead to increased cytotoxicity, incorporation or immobilization of the vector into or onto the material offers a promising platform for localized and sustained gene delivery^[130]^. Hao *et al.*^[132]^ developed a dual-gene therapy factor delivery system based on chemical vapor deposition on a PCL/PLGA/titanium surface, all of which are FDA-approved biomaterials. This system successfully delivered BMP-7 and PDGF-B gene vectors to human PDL cells, resulting in highly localized and sustained protein production (peak expression at day 7–10) compared to direct physical absorption. An alternative approach to incorporating gene therapy vectors into biomaterials was reported by Gonzalez-Fernandez *et al.*^[133]^ where peptide-based plasmid DNA encoding BMP-2 and SRY-Box Transcription Factor 9 (SOX-9) was incorporated into tunable hydrogels, which could direct either rapid and transient, or slower and more sustained transfection of host cells within a spatiotemporally defined pattern. *In vivo*, the construct supported the development of vascularized bony tissues overlaid by a layer of stable cartilage. Clinical trials are needed to assess the safety and efficacy of gene therapy for periodontal tissue engineering and bone regeneration purposes in humans - to the best of our knowledge, these studies have not yet been conducted.

### Cell therapy

Cell therapy can be defined as the treatment of disease by introducing new cells into a tissue^[134]^. Cell-based tissue engineering is one of the most frequently used tissue engineering methodologies for periodontal regeneration in the literature and is associated with distinct advantages^[135]^. These include the direct placement of cells and secreted growth factors into a periodontal defect, which significantly reduces the expected lag phase for progenitor cell recruitment to the defect site. The most widely used cell types include PDL cells, BMSCs, and dental pulp stem cells (DPSCs). A specific subset of CD90^+^ and CD14^+^ BMSCs and monocyte progenitors have been demonstrated to exhibit strong osteogenic potential and can be isolated and expanded from a small sample of autologous bone marrow and then delivered back to the patient. This method allows localized delivery of patient-specific cells and has been shown in phase I/II trials to increase bone formation^[136,137]^.

However, several obvious disadvantages exist, including the need to harvest human stem cells, the weak mechanical stability of transplanted cell sheets and unpredictable outcomes for stem cell-scaffold combinations. While PDL cells can easily be isolated from the root surface after tooth extraction, or the alveolar bone surface of an extraction socket, BMSCs and DPSCs are much more difficult to access from human patients. Cell-sheet engineering represents a relatively non-invasive technology incorporating specialized substrates allowing easy detachment of expanded cell sheets while also maintaining an intact extracellular matrix. However, it is difficult to achieve biomechanical fixation utilizing this approach. The cell sheets may easily become displaced during suturing or mastication, resulting in impaired regenerative outcomes^[138]^. The concurrent use of a scaffold can provide support for the cell sheet and maintain the necessary space for tissue regeneration^[104]^. However, Yan *et al.*^[139]^ investigated the periodontal regenerative capacity of a chitosan hydrogel with or without cell loading, and reported no differences in new bone formation, new ligament formation and epithelial downgrowth. Yu *et al.*^[140]^ found that implanted PDL cells were not integrated into the newly-formed bone area, but still managed to stimulate osteogenic activity in the surrounding host cells through an indirect manner.

The feasibility of implementing cell therapy through cell-sheets or stem cells in combination with scaffolds should be validated in future studies to investigate the underlying cellular signaling pathways and the regenerative capacity of these approaches^[141]^. In relation to the use of biomaterials that have been demonstrated to provide relatively predictable regenerative outcomes, cell therapy represents a comparatively complex approach for implementation in everyday clinical practice^[142]^. Developing methods of improving the clinical translatability of cell therapeutic approaches must be considered in future studies. The targeted recruitment of host cells through the development of cell-homing scaffolds that do not require cell transplantation has strong potential to circumvent some of the limitations associated with cell delivery^[143]^.

### Cell free therapy: extracellular vesicles for periodontal and bone regeneration

The utility of MSCs for tissue engineering purposes is based not only on their ability to differentiate within damaged tissues into specialized cells, but also on their paracrine activity^[144]^. In fact, research has shown that only a small portion of MSCs delivered systemically or locally are physically incorporated into target tissues^[145,146]^. This data suggests that their regenerative potential is likely mediated through indirect mechanisms linked to growth factor and extracellular vesicle secretion.

MSC conditioned media has recently emerged as a rich source of growth factors and cytokines that has potential applications towards cell-free regeneration of bone^[147]^. Extracellular vesicles secreted from MSCs have also garnered interest in periodontal tissue engineering due to their unique capacity to transfer proteins, lipids and various forms of RNA to surrounding cells in order to mediate a wide variety of biologic functions. Extracellular vesicles clearly contain a bewildering array of biologic molecules; analysis of the proteome of MSC-derived extracellular vesicles identified 730 different proteins. These included surface receptors and signaling molecules involved in self-renewal and differentiation of MSCs, as well as in cell proliferation, adhesion, migration and morphogenesis of many other cell types^[148]^. Although the physiologic role of MSC-derived extracellular vesicles is currently not fully understood, preclinical and clinical trials have demonstrated promising results in the context of periodontal and bone regeneration^[149–151]^, suggesting that MSC-derived extracellular vesicles possess many of the regenerative properties of their cellular counterparts.

BMSC-derived small extracellular vesicles were recently shown to promote migration, proliferation and osteogenic differentiation of human PDLCs *in vitro,* and to mitigate periodontal bone loss, inflammatory infiltration and collagen degradation in rats with ligature-induced periodontitis^[152]^. Shen *et al.*^[153]^ demonstrated that DPSC-derived exosome-incorporated chitosan hydrogel accelerated alveolar bone healing in a murine periodontitis model, suppressing inflammation and stimulating macrophage polarization towards an anti-inflammatory phenotype. Interestingly, the immunomodulatory effects on macrophage polarization were linked to functional mi-RNA contained within the exosomes. In another study, Chew *et al.*^[154]^ demonstrated that MSC-derived exosomes delivered via a collagen sponge promoted regeneration of surgically induced periodontal defects in a rat model.

Although a large body of research has focused on MSC-derived extracellular vesicles, it is important to realize that many, if not all cells, secrete micro- and nano-vesicles which are believed to play important roles in intercellular communication at both local and distant levels^[155]^. Part of the complexity in assessing the influence of extracellular vesicles on periodontal and bone regeneration lies in identifying the active components out of a highly heterogeneous mixture of molecules. Additionally, it is unclear how cell culture conditions may influence the composition and yield of exosomes^[156]^, leading to questions surrounding consistency and repeatability of clinical outcomes. Future research is needed in the form of clinical trials to verify whether cell-free approaches can attain comparable outcomes to cell therapeutics.

## CONCLUSION

Many key advancements have occurred over the last 10 to 20 years in the field of periodontal tissue engineering and regenerative medicine. In the future, it may be possible to mimic natural healing processes by developing biomimetic scaffolds that deliver biologics in response to microenvironmental stimuli and cellular demands. 3D-printing of entire organs is another exciting future possibility. Despite these developments, much work remains to be done to enable clinical translation. Future studies are needed to confirm the specific tuneable concentrations of growth factors which yield the highest regenerative outcomes, and to better delineate the differential effects of growth factors on the various hard and soft tissue components comprising the periodontal and peri-implant support. In addition, future research should further investigate the validity of tissue engineering-based approaches implementing combinations of biologics as well as sequential and controllable delivery. The development of interconnected and highly porous degradable scaffolds with appropriate biomechanical properties will allow for more favourable cell-ECM interactions. Regarding cell therapy, future work should focus on improving the viability, stability and regenerative functions of implanted cell sheets. Ultimately, oral tissue engineering-based technologies have a common aim - to provide patient-specific treatment options that maximize function, esthetics and the quality of patient care.

## Figures and Tables

**Table 1.** Summary of commercially available and commonly studied biologic agents^[17,26,66]^

	EMD	PDGF-BB	FGF-2	BMP-2 and -7
Endogenous sources	Hertwig’s epithelial root sheath	BB isoform: osteoblasts, macrophages & endothelial cells. Only AB isoform is derived from platelets and found in blood.	Macrophages & endothelial cells	Osteoblasts & bone matrix
Composition	> 90% amgelogenin, Small % ameloblastin, fetuin A & α-1-antichymotrypsin	Protein	Protein	Protein
Mechanism of action	Exact MOA unknown. Believed to play a role in cementogenesis.	↑ chemotaxis of PMNs & monocytes, ↑ endothelial cell chemotaxis, proliferation & differentiation, ↑ fibroblast proliferation & ECM synthesis	↑ fibroblast proliferation & ECM synthesis ↑ endothelial cell chemotaxis, proliferation & differentiation ↑ mesenchymal progenitor cell migration	BMP-2: ↑ mesenchymal osteoprogenitor cell migration BMP-7: ↑ osteoblast and chondroblast differentiation
FDA approval (Labelled usage)	Yes (intrabony, class 2 furcation defects & gingival recession coverage)	Yes (intrabony defects, furcations, gingival recession)	No	BMP-2: Yes (sinus augmentation, socket preservation) BMP-7: No for dental usage; approved for spinal fusion & long bone non-union treatment
Preclinical and clinical evidence for other usages	Clinical evidence for treatment of peri-implantitis-associated defects^[20–22]^	Clinical evidence for GBR^[41,149]^ and peri-implantitis-associated defects^[150]^	Clinical evidence for intrabony defects^[57]^; pre-clinical evidence for peri-implant defects^[151]^	Clinical evidence for GBR and pre-clinical evidence for peri-implantitis-associated defects^[67,77]^
Commercial products	Emdogain (Straumann)	GEM 21S (Lynch Biologics)	None yet	BMP-2: Infuse Bone Graft (Medtronic) BMP-7: Osigraft (Stryker Biotech)

↑: increased; EMD: enamel matrix derivative; rhPDGF-BB: recombinant human platelet-derived growth factor-BB; FGF: fibroblast growth factor; BMP: bone morphogenetic protein; PMN: polymorphonuclear leukocyte; ECM: extracellular matrix; FDA: Food and Drug Administration; GBR: guided bone regeneration

**Table 2.** Summary of current advancements in multiphasic scaffolds

Component	Fabrication technique	Experimental model	Outcomes	Literature support
PCL	Solution electrospinning and fused deposition modeling	Athymic rats: ectopic model (subcutaneous implantation of scaffold-dentin slide complex)	Higher rate of cementum-like tissue deposition at the dentin-cell sheet interface was observed. However, there was poor integration of new PDL-like tissue with the bone compartment	Vaquette *et al.*^[111]^
Chitin-PLGA + bioactive glass	Freeze lyophilization	Rabbits: maxillary periodontal defects	Formation of new cementum, fibrous PDL and alveolar bone were observed with well-defined bony trabeculae after 3 months. However, the thickness and shape of the scaffold could not be customized.	Sowmya *et al.*^[112]^
PCL	Melt electrospinning and solution electrospinning	Sheep: periodontal dehiscence defects	Excellent tissue integration between the bone and PDL compartments as well as the root surface was observed. Constructs combined with PDLSCs showed greater bone fill at week 10 compared with BMSCs and gingival cells.	Vaquette *et al.*^[113]^

PCL: polycaprolactone; PDL: periodontal ligament; PLGA: poly(lactic-co-glycolic acid); BMSCs: bone marrow mesenchymal stem cells; PDLSC: periodontal ligament stem cells

**Table 3.** Summary of current advancements in 3D-printed scaffolds

Component	Fabrication technique	Experimental model	Outcomes	Literature support
PCL + polyglycolic acid	3D wax printing	Immunodeficient rats: surgically created periodontal defects	More physiologic PDL-like fiber organization was demonstrated for fiber guiding scaffolds compared to random scaffold architectures	Park *et al.*^[116]^
PCL + hydroxyapatite	Layer-by-layer deposition	Immunodeficient mice: ectopic model (subcutaneous implantation)	The delivery of biologic cues combined with the seeding of DPSCs led to the formation of bone, PDL and cementum/dentin-like tissues in the various compartments, and inserting PDL fibers with a perpendicular orientation were observed	Lee *et al.*^[117]^
PCL	Fused deposition modeling	Human study: Pilot randomized controlled clinical trial	Insertion of PCL scaffolds in fresh extraction sockets resulted in normal bone healing and less vertical ridge resorption after 6 months compared to spontaneous healing	Goh *et al.*^[118]^
PCL	Selective laser sintering	Human study: aggressive periodontitis	The construct remained intact for 12 months following therapy, but became exposed after 13 months	Rasperini *et al.*^[119]^
PCL	Layer-by-layer deposition	Human study: posterior mandibular defects	A straightforward and reproducible workflow for fabrication of highly porous (84% porosity) custom 3D-printed scaffolds for large volume alveolar bone regeneration was reported	Bartnikowski *et al.*^[120]^

PCL: polycaprolactone; PDL: periodontal ligament; DPSCs: dental pulp stem cells

**Table 4.** Summary of current advancements in gels and hydrogels

Component	Fabrication Technique	Experimental Model	Outcomes	Literature support
Calcium phosphate cement + propylene glycol alginate		Nonhuman primates: Three-wall intrabony defects	The experimental group showed significantly less epithelial downgrowth and enhanced cementum + PDL regeneration compared to the control	Wang *et al.*^[122]^
GelMa + PDLSC	3D Bioprinting	*in vitro*	3D bioprinting conditions for attaining high resolution, dimensional stability and cell viability of periodontal ligament cells were optimized	Thattaruparambil Raveendran *et al.*^[125]^
Polyisocyanopeptide + PLGA	Electrospraying	Rat: ectopic model (subcutaneous implantation)	This gel system exhibited tunable drug release, optimal injectability, long-term structural stability and no obvious *in vivo* inflammatory response	Wang *et al.*^[124]^

PDL: periodontal ligament; PLGA: poly(lactic-co-glycolic acid); PDLSC: periodontal ligament stem cells; GelMa: gelatin methacryloyl

## Data Availability

Not applicable.
